# Maternity Homes in Industrial Life

**Published:** 1914-05-02

**Authors:** 


### MATERNITY HOMES IN INDUSTRIAL LIFE.

### The State and the Environment of Pregnancy.

Some few years ago a vivid light was thrown upon the conditions in which "slum" babies are brought up by *Sixpenny Pieces*. Those medical men who had ever practised in districts such as the poorer parts of South London—in which the author's scene was laid—knew that there was little of exaggeration in the description of a confinement which was given. Horrible as some of the incidents narrated were, there were an optimism, a humour, a capacity for seeing the bright side without drawing a veil over the dark one in the book which mitigated the first feelings of repulsion. Thus by its means the reading public was given an insight into a side of life which otherwise would have remained beyond ken; and it cannot be doubted that the conscience of that public was distinctly stirred.

There have been, of course, other factors forcing interest in problems of the kind on the educated classes. Many societies and many individuals have done their share in this work; and it is from no want of appreciation of their labours that they are not now particularised by name. Suffice it that the general opinion is that in the environment of the puerperal woman of the poorest classes there is ample room for, and urgent need of, improvement. As to the exact measures best calculated to remedy the evils in question, there is less unanimity.

State aid, in some form or another, is the foundation of most of them; and it is quite true that there are few questions of the day in which the State is more vitally concerned, or has a better right of interference and control. State aid, however, means that schemes projected in which such aid is invoked must be as inexpensive as possible; for it is certain that any scheme of State aid must apply to the bulk of our population, and that the cost of any elaborate scheme of care, applied on such a large scale, would be prohibitive. For this reason we take leave to doubt whether the suggestions put forward by Mr. Frank Webb will fructify, attractive though they look on paper. Pending State aid, these suggestions are put forward for the consideration of philanthropic persons, municipal authorities, and the Insurance Commissioners. They include the provision, in suitable localities, of places, "which need not be large or expensively equipped, to which expectant mothers could go if they wished for the period of their confinement, and so avoid the distress, anxiety, and worry, as well as the discomfort and neglect, which such an event in the small cottage-house is apt to entail."

In the cities and large towns, as Mr. Webb observes, some such cases are provided for by lying-in hospitals. As a general rule these are the cases in which the need of medical skill, rather than straightforward nursing, has been the predominant factor in the case. Obstetric difficulties or intercurrent acute diseases are thus fairly well provided for, together with a small percentage of the much more numerous normal cases; all this, of course, holds good for a few large towns only, or rather for portions of them. No such provision is made for industrial mothers as a class.

It is impossible to doubt that maternity homes on these lines, no matter by what agency provided, would be a great blessing to the poorer classes. Not only would they reduce suffering and lead to speedier and surer convalescence for the mothers, but they would also do something to reduce the high infantile mortality among the urban proletariat. Baby-management and hygiene would be inculcated by example and precept, to the great advantage of succeeding generations. Certain obvious weaknesses about the idea, such as the objection of the mother to leave her home and her family, could probably be surmounted somehow, though the difficulty on this score might prove greater than Mr. Webb anticipates. The serious flaw, to our mind, is the financial burden which so comprehensive a scheme would throw on the taxpayer. This, we fear, foredooms at present, and for years to come, the projects which Mr. Webb has outlined. It is easy enough to call the homes "inexpensive," but in practice we fear they would not prove to be so, either to start or maintain.

The question of a Ministry of Public Health has also been once more agitated in the course of the discussions which have lately been taking place. This is a reform which is, in our opinion, inevitable and highly necessary. It is not likely that many years more of the present makeshift arrangements, whereby three or four State departments deal with fragments of the public health of the country, can continue. Our one misgiving about the Ministry of Public Health is lest the blight of party politics should attack the public-health organisation. It would be almost better to muddle along in our present slipshod way than to have the party caucus and the paid politician meddling with the health of the nation: the Insurance Act is sufficient instance of the disastrous results of trying to make a party "score" out of such legislation as that for which the Ministry of Public Health would be responsible. Again, there would be grave disadvantages about the selection of the Minister on the usual principles which guide party managers in making these appointments. Faithful party service should, though we fear it would not, be regarded as a disqualification. Until a much larger number of medical men enter Parliament than has ever been the case hitherto, we doubt whether there would be sufficient choice (on either side of the House) for a really suitable Minister to be found. (We are assuming that only medical men would be eligible for selection; this restriction we regard as essential.) Certainly in the present House we cannot think of anyone fit for the post.

